# Molecular Aspects in Porous Silicas Related to Adsorption and Catalytic Processes

**DOI:** 10.3390/ijms25137085

**Published:** 2024-06-27

**Authors:** Juan Antonio Cecilia, Ramón Moreno-Tost

**Affiliations:** Departamento de Química Inorgánica, Cristalografía y Mineralogía, Universidad de Málaga, 29071 Málaga, Spain

The aim of this special issue is to show the advances in the different applications that inorganic materials based on silica have had in recent years. Despite being such a simple solid, with only Si and O in its composition, and so abundant in nature, we could say that it has given rise to its own line of research since Stöber [1] first prepared spherical SiO_2_ nanoparticles. Subsequently, researchers at Mobil [2] developed the M41S family of mesoporous silica solids from which many other families of silicas with different structures, morphologies and sizes have been developed and have found applications ranging from adsorption to medicine [3].

Porous silicas can modulate their particle size to generate micro-, meso- or macroporous materials depending on their use or application. The modulation of the porosity provides many advantages to these porous structures as ordered structures, regular and uniform channels, adjustable pore diameter and pore volume, large surface area, high thermochemical stability and potential functionalization of its surface. Taking into account these excellent properties, porous silicas have been widely applied in adsorption [4,5], catalysis [6,7], separation processes [8,9], gas storage [10,11], medical treatments [12,13], biomedicine [14] and drug release [15,16].

Ordered porous silicas are generally formed with a micelle in the form of surfactant, which acts as structure directing agent [17,18]. Then, the silica grows around this template. Then, the surfactant, which is generally an organic structure, is removed by calcination or extraction, forming an amorphous silica with interconnected channels of adjustable dimensions depending on its application. This allows accommodation from macromolecules, such as biomolecules, to small molecules. In the same way, the presence of high density of silanol groups [19] in the walls of the amorphous silica also allows modify the surface of the porous silica with other functional groups to acquire new properties and applications [20].

With the papers accepted in this special issue, we have tried to give an insight into some of the possibilities offered by silica and to discover for potential readers new advanced applications of silica and its solid derivatives.
